# Investigating the Nutritional and Sensory Potential of Selected Indigenous South African Fruits: Physicochemical Properties, Jam Production and Quality Evaluation

**DOI:** 10.1002/fsn3.70287

**Published:** 2025-05-19

**Authors:** Karen de Jager, Wilma Augustyn, Thierry Regnier, Belinda Meiring

**Affiliations:** ^1^ Agricultural Research Council‐Tropical and Subtropical Crops Agro‐Processing Nelspruit South Africa; ^2^ Department of Biotechnology and Food Technology Tshwane University of Technology Pretoria South Africa; ^3^ Department of Chemistry Tshwane University of Technology Pretoria South Africa

**Keywords:** jam production, physicochemical analysis, phytochemical content, sensory properties, shelf life, South African indigenous fruits

## Abstract

South African indigenous fruits remain largely underutilized, with limited research conducted on their potential for developing novel products. Indigenous fruits have unique appearances, flavors, and nutritional values. However, these fruits have a short fruit season. Processing and new product development can ensure longer shelf life and income generation through value‐adding. This study aimed to evaluate the potential of underutilized indigenous fruits *Carissa edulis* (simple‐spined num‐num), *Dovyalis affra* (Kei‐apple), *Dovyalis longispina* (Natal apricot), *Englerophytum magalismontanum* (stamvrug), and *Sclerocarya birrea* (marula) for jam production. Due to genetic variation among Kei‐apple fruits, two selections were included in the study. The study also investigated the impact of storage time and temperature on the quality of the jams. Physicochemical analyses were conducted on the fruit pulp, freshly prepared jams, and stored jams. Consumers preferred jams with a higher Total soluble solid/Titratable acid ratio, such as the num‐num, stamvrug, and marula, with 64%, 70%, and 60% of the panelists indicating they would buy the jam. Jam color stability was greater when stored at 25°C than at 35°C. Num‐num and stamvrug jams exhibited the best color retention, even after 6 months of storage at 25°C, with Delta E values of 1 and 4, respectively. Indigenous fruit jam processing, storage time, and temperature altered vitamin C, phenolic, flavonoid, tannin, and lycopene contents. Kei‐apple 103 jams retained a high vitamin C content of 181.30 mg/100 g after 6 months of storage at 25°C. The Natal apricot demonstrated the highest lycopene content (39.23 mg/100 g) when stored at 35°C for six months and a substantial total carotene content of 23.37 mg/100 g, even under high‐temperature conditions. Marula jam outperformed other indigenous fruit jams in retaining phenolics and flavonoids, with values of 3.47 mg GAE/g and 1.9 mg CE/g, respectively. Meanwhile, the tannin content of num‐num and stamvrug jams stored at 35°C remained significant, measuring 6.17 mg and 6.67 mg cyanidin chloride/g, respectively. This study indicated considerable variation in physicochemical composition between Kei‐apple selections. Future research on the effect of genetic variation of other indigenous fruits should also be included. Additionally, product development using Natal apricot and Kei‐apple selections with extremely high acidity could focus on creating mixed fruit jams by incorporating other tropical fruits to enhance sensory appeal and achieve greater consumer acceptance. The indigenous fruit jams evaluated in this study demonstrate potential for development as niche products, offering opportunities to support income generation for small‐scale processing enterprises.

## Graphical abstract

High‐quality indigenous fruit jam can be prepared and stored at 25°C for 60 days with minimal quality degradation. All evaluated indigenous fruit jams exhibit potential for commercial development and niche market sales.

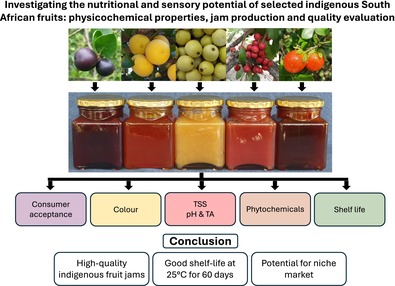

## 1 Introduction

African indigenous fruit trees are rich sources of vitamins, minerals, protein, and phytochemicals (Omotayo and Aremu 2020) and contribute to the livelihoods of rural households (Omotayo and Aremu 2020) and contribute to the livelihoods and income generation of rural households (Kalaba et al. 2009; Mng'omba et al. 2008; Motlhanka et al. 2008; Ræbild et al. 2011; Stadlmayr et al. 2013; Bille et al. 2013; Kalaba et al. 2009). The fruits are highly nutritious and contain significant quantities of antioxidants (Kucich and Wicht 2016; Loots et al. 2006; Motlhanka et al. 2008; Sibiya et al. 2020). These plants play an important role in food security by providing nutrients when other food sources are unavailable. Indigenous fruits are seasonal and highly perishable. Processing these fruits can extend their shelf‐life and ensure a year‐round food supply (Brandão et al. 2018; Dlamini and Solomon 2019; Maroyi 2011; Wynberg et al. 2003). This can also lead to higher income due to value‐adding. The commercialization of indigenous fruits has been neglected in the past (Kalaba et al. 2009) due to a perception that these fruits are of low standard and a lack of knowledge about their value‐adding potential (Onomu 2023). Few African fruit products have been developed. Amarula liqueur is a very well‐known example of an African product. According to Saka et al. (2008), there is a need to develop processing techniques and diversify the products from indigenous fruits. Indigenous fruits are characterized by their vibrant colors, unique tastes, distinct flavors, and exceptional nutritional properties, offering great potential for innovative product development. Jams concentrate the natural flavors of these fruits, often enhancing their sensory appeal. Product development of South African indigenous fruits can appeal to diverse consumer preferences. Fruit jam is a low‐cost processing method to preserve fruit to ensure an extended shelf life. Sensory evaluation is a crucial part of any product development to determine the consumer acceptability of the products. No information is currently available in the literature on South African indigenous fruit jams, particularly their sensory or physicochemical properties and storage stability. Hence, the objective of this study was to evaluate the potential of five indigenous fruits, *Carissa edulis* (simple‐spined num‐num), *Dovyalis affra* (Kei‐apple) (Formerly *Dovyalis caffra* ), *Dovyalis longispina* (Natal apricot), *Englerophytum magalismontanum* (stamvrug), and *Sclerocarya birrea* (marula) for jam production. These jams were stored for 3 and 6 months at 25°C and 35°C. The effects of storage time and temperature on the color, pH, Titratable acid (TA), Total Soluble Solids (TSS), and phytochemical composition were assessed.

## 2 Materials and Methods

### 2.1 Sample Collection

*Carissa edulis* (simple‐spined num‐num), *Dovyalis affra* (Kei‐apple), *Dovyalis longispina* (Natal apricot), *Englerophytum magalismontanum* (stamvrug) and *Sclerocarya birrea* (marula) were used for jam‐making. Two selections of *Dovyalis affra* (Kei‐apple) were compared for their suitability for jam‐making, since many Kei‐apple selections are highly acidic, rendering them unsuitable for fresh consumption. Fruits were harvested from the Agricultural Research Council–Institute for Tropical and Subtropical Crops (ARC‐TSC) miscellaneous orchards (Mpumalanga, South Africa). Mature green marula fruits were collected from the ground, while all the other selected indigenous fruits were harvested from the tree when ripe, based on color. Since the fruits do not all ripen simultaneously, they were stored at room temperature until ripe and then transferred to a cold room at 10°C until all fruits reached optimum ripeness for processing. The decayed fruits were discarded, and the remaining fruits were washed and pulped using a mechanical pulper (Dryers for Africa, Mbombela, South Africa).

### 2.2 Preparation of Jams

Fruit pulp was used for jam‐making. Standard jam‐making methods were used as described by Mayhew (2009). All jams were prepared with a 1:1 pulp‐to‐sugar ratio, except for the Kei‐apple 103, which required a 1:1.25 pulp‐to‐sugar ratio due to the fruit's excessive acidity, even when fully ripe. Strict hygiene practices were followed to ensure the microbial load of the product was kept sufficiently low. The hot jam was poured into sterilized glass jars, sealed, and turned upside down to sanitize the lid and headspace. The jars were flipped over after a few minutes.

### 2.3 Methods of Analysis

#### 2.3.1 Sensory Evaluation

##### 2.3.1.1 Visual Acceptance Test

**FIGURE 1:**
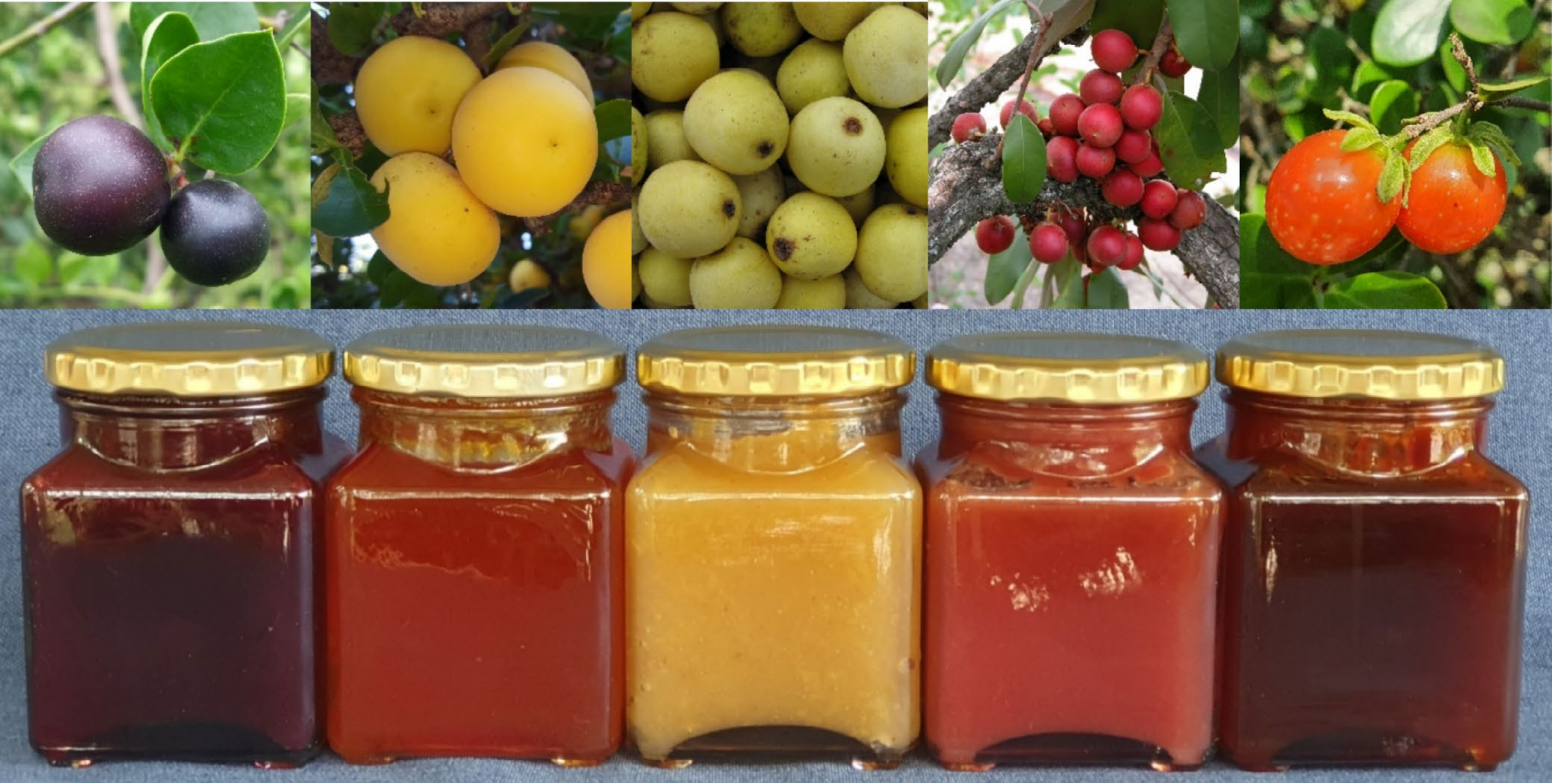
Photographs of indigenous fruit (Top) and their respective jams (Bottom) were used for consumer acceptance tests. Left to right: Num‐num, Kei‐apple, marula, stamvrug, and Natal apricot.

Given that indigenous fruit jams may be unfamiliar to consumers, and they might rely on the image and color of the jams as a purchasing guide, a picture of the fruit was placed on the jar for the consumer acceptance test. Consumers (*n* = 98) were asked to rank their preferences for the jams based on the color of the jams and photos of the fruit (Figure 1). The total score for each jam was calculated according to the preferences indicated by the panelists, with the 1st choice receiving 5 points, the 2nd choice 4 points, the 3rd choice 3 points, the 4th choice 2 points, and the 5th choice 1 point. The percentages for each jam score were then calculated.

##### 2.3.1.2 Hedonic Test

Sensory evaluations were done on all the jams according to the guidelines provided by Kemp et al. (2011) to determine the most preferred jams. The panelists were between 18 and 70 years old, non‐smokers, with no tasting impairments or health issues. Forty‐seven panelists participated in this experiment, with 55% being female. Each untrained panelist signed a consent form before participating to indicate their acceptance to participate in the study. Three‐digit coded samples were used, and the jams were presented in a completely random order. Water was used between samples to clear the palate. The jams were evaluated using a 9‐point hedonic scale (Kemp et al. 2011) to determine acceptance, i.e., each jam was evaluated for color, smell, taste, mouth feel, and overall impression using a scale of 1 to 9 (1 = dislike extremely and 9 = like extremely). The consumers also indicated their willingness to buy the jams.

#### 2.3.2 Shelf‐Life

Centrifuge tubes were filled with samples of the fresh fruit pulp and jams of the respective indigenous fruits and immediately frozen at −20°C for further analyses. In addition, all jams were stored at 25°C and 35°C for 3 and 6 months and then transferred to centrifuge tubes before freezing at −20°C until conducting further analyses. The storage temperatures of 25°C and 35°C were chosen to simulate typical ambient conditions in South Africa, particularly in Mpumalanga, where average summer temperatures range from 20°C to 30°C, with occasional peaks above 35°C (South African Weather Service 2024). The storage temperatures were chosen to reflect moderate (25°C) and elevated (35°C) ambient storage scenarios, enabling an evaluation of jam quality under conditions representative of real‐world environments without cooling facilities. The effect of storage time and temperature on the color, TSS, pH, TA, and phytochemicals was evaluated.

#### 2.3.3 Color

The color attributes of each sample were determined using a handheld colourimeter (Lovibond, UK), based on the CIELAB color space (L*, a*, b*). The L* (Lightness), a* (red‐green), and b* (yellow‐blue) values were recorded (Bicanic et al. 2010; Kaushik et al. 2014; Siddiq et al. 2013). The samples were homogeneously distributed in a cuvette and analyzed in an enclosed stand (Lovibond, UK) with a white background. All sample colors were measured in triplicate. The total color difference (∆E) between the fresh and stored jams was calculated using the equation (Liu et al. 2014):

$$∆E={\left(∆L^{2}+∆a^{2}+∆b^{2}\right)}^{0.5}$$

#### 2.3.4 Total Soluble Solids (TSS)

The Total Soluble Solids (TSS) of the pulp and jam samples were measured using a digital Pocket Pal handheld refractometer (RX 5000, Atago CO. Ltd., Tokyo, Japan).

#### 2.3.5 Titratable Acids (TA) and pH

The pH and titratable acidity (TA) of the pulp and jams were measured using an automatic titrator (Steroglass Flash Autosampler AS24, Perugia, Italy). The samples were prepared by adding 80 mL of distilled water to 5 mL of the sample. The titration was performed using NaOH (0.1 M) to a pH endpoint of 8.2 (Soppelsa et al. 2023). The TSS/TA ratio was then calculated.

### 2.4 Determination of Phytochemicals

#### 2.4.1 Vitamin C

The vitamin C content was determined using a method described by Odriozola‐Serrano et al. (2007) with slight modifications. Fruit pulp and jam material (0.2 g) was extracted using 10 mL of 4.5% metaphosphoric acid. The mixture was sonicated in an ultrasonic bath containing ice‐cold water for 30 min before filtration. The prepared samples were then analyzed using a Shimadzu HPLC (LC‐2030C 3D, Shimadzu Corporation, Kyoto, Japan) equipped with a C18 Luna column (150 mm, 4.6 mm, 5 μm) at 25°C. Water: acetonitrile: formic acid (99:0.9:0.1 v/v/v) was used as the mobile phase at a flow rate of 1 mL/min in isocratic mode, with an injection volume of 20 μL and a detection wavelength of 245 nm. Ascorbic acid prepared at different concentrations was used as a standard for the preparation of a calibration curve. The vitamin C content was expressed as mg of Vitamin C/100 g dry weight (DW) of the sample.

#### 2.4.2 Lycopene

The method described by Nagata and Yamashita (1992) was used to determine the Lycopene content of the fruit pulp, fresh jams, and jams after storage. The freeze‐dried sample (100 mg) was vigorously shaken with 10 mL of acetone‐hexane mixture (2:3) for 1 min and filtered through the Whatman No. 4 filter paper. The absorbance of the filtrate was measured at 453 nm, 505 nm, 645 nm, and 663 nm. Lycopene was calculated using the equation:

$$\begin{array}{cc} \text{Lycopene}\;\left(\frac{\mathrm{mg}}{100}\mathrm{mL}\right)=\; & -\left(0.0458\times 663\;\mathrm{nm}\right)+\left(0.204\times 645\;\mathrm{nm}\right)\\ & +\left(0.372\times A_{505}\;\mathrm{nm}\right)-\left(0.0806\times A_{453}\;\mathrm{nm}\right) \end{array}$$

The assays were carried out in triplicate, and the results were average mean values expressed as mg of lycopene/100 g of DW.

#### 2.4.3 Total Carotenoids

The total carotenoids were determined using a similar method to the lycopene analysis. Samples were read at 450 nm, and the total carotenoid content was calculated using the equation described by de Carvalho et al. (2012):

$$\text{Carotenoids content}\;\left(\frac{\mathrm{\mu g}}{g}\right)=\frac{\left(A\times V\;\left(\mathrm{mL}\right)\times 104\right)}{\left(A1\%1\;\mathrm{cm}\times P\left(g\right)\right)}$$

where *A*, Absorbance; *V*, Total extract volume; *P*, sample weight; *A*1% 1 cm = 2592 (β‐Carotene Extinction Coefficient in Hexane).

#### 2.4.4 β‐Carotene

β‐carotene content was determined using the method described by Biehler et al. (2010) with slight modifications. The procedure was carried out in the dark to avoid the effect of light on β‐carotene. Methanol (5 mL) was added to a 0.2 g dry sample, and the mixture was vortexed for 10 s. Thereafter, 15 mL hexane: acetone (1:1 v/v) solution was added and vortexed for 10 s before sonicating in an ultrasonic bath containing ice‐cold water for 15 min. Saturated sodium chloride solution (5 mL) was added to the mixture, vortexed for 10 s, and centrifuged at 1073.3 *g* for 2 min. The collected supernatant was filtered through a 0.45 mm syringe filter before analysis using a Shimadzu HPLC (LC‐2030C 3D, Shimadzu Corporation, Kyoto, Japan) equipped with a C18 Luna column (150 mm 4.6 mm, 5 μm) at 35°C. Acetonitrile: dichloromethane: methanol (70:20:10, v/v/v) was used as a mobile phase at a flow rate of 1 mL/min in isocratic mode, with an injection volume of 20 μL and a detection wavelength of 450 nm. Identification and quantification of β‐carotene were achieved by plotting a calibration curve using a β‐carotene standard. The β‐carotene content was expressed as mg β‐carotene/100 g DW of the sample.

#### 2.4.5 Total Phenolic and Flavonoid Content

Phenolic and flavonoid extractions were performed using the method described by Amoo et al. (2012). Pulp and jam samples (0.2 g) were extracted with 10 mL of 50% MeOH for 30 min in an ultrasonic bath containing ice‐cold water. The samples were centrifuged at 2000 rpm for 2 min. Total phenolic content was determined using the Folin‐Ciocalteau method (Du Plooy et al. 2009) with modifications. A reaction mixture containing 50 μL of the sample extract, 450 μL distilled water, 250 mL of Folin‐Ciocalteau reagent, and 1250 μL NaHCO_3_ (2% w/v) was briefly vortexed and incubated for 40 min at room temperature. The absorbance was then recorded using a spectrophotometer (Specord 210 plus, Analytik Jena, Jena, Germany) at 725 nm. The assay was done in triplicate, and a calibration curve was prepared using gallic acid as a standard. Results were expressed in mg gallic acid equivalent (GAE) per gram DW.

Flavonoid content was determined using an aluminium chloride method (Ribarova et al. 2005), with modifications. A reaction mixture containing 250 μL of the sample, 1.6 mL of distilled water, 75 μL of 5% w/v sodium nitrite, 75 μL of 10% w/v aluminium chloride, and 0.5 mL of 1 M NaOH was briefly vortexed, and the absorbance was measured at 510 nm. The assay was done in triplicate, and a calibration curve was prepared using catechin as a standard. Results were expressed in mg catechin equivalent (CE) per gram DW.

#### 2.4.6 Condensed Tannin (Proanthocyanidin)

The condensed tannin (proanthocyanidin) content was determined using the butanol‐HCl method as described by Makkar (2003). Three milliliters of butanol‐HCl (95:5 v/v) were added to 500 μL of each sample, followed by 100 μL of ferric reagent (2% w/v ferric ammonium sulphate in 2 N HCl). The mixtures were placed in a boiling water bath for 60 min. Each sample was then transferred into a cuvette and absorbance read at 550 nm using a UV–Visible spectrophotometer (Varian Cary 50, Australia) against a blank prepared similarly but without heating. Each sample had three replicates. Condensed tannins (%dry matter) expressed as leucocyanidin equivalents were calculated using the equation described by Porter et al. (1985):

$$\begin{array}{c} \text{Condensed tannins}\;\%\;\mathrm{dry}\;\text{matter}=A_{550}\;\mathrm{nm}\times 78.26\\ \times \text{Dilution factor}\;\%\;\mathrm{dry}\;\text{matter} \end{array}$$

where *A*_550_ nm is the absorbance value at 550 nm.

### 2.5 Statistical Analysis

The results were subjected to analysis of variance (ANOVA). Mean values were compared using Fisher's protected least significant difference (LSD) test at a 5% significance level, utilizing Genstat 22.1 software.

## 3 Results and Discussion

### 3.1 Sensory Evaluation

**FIGURE 2:**
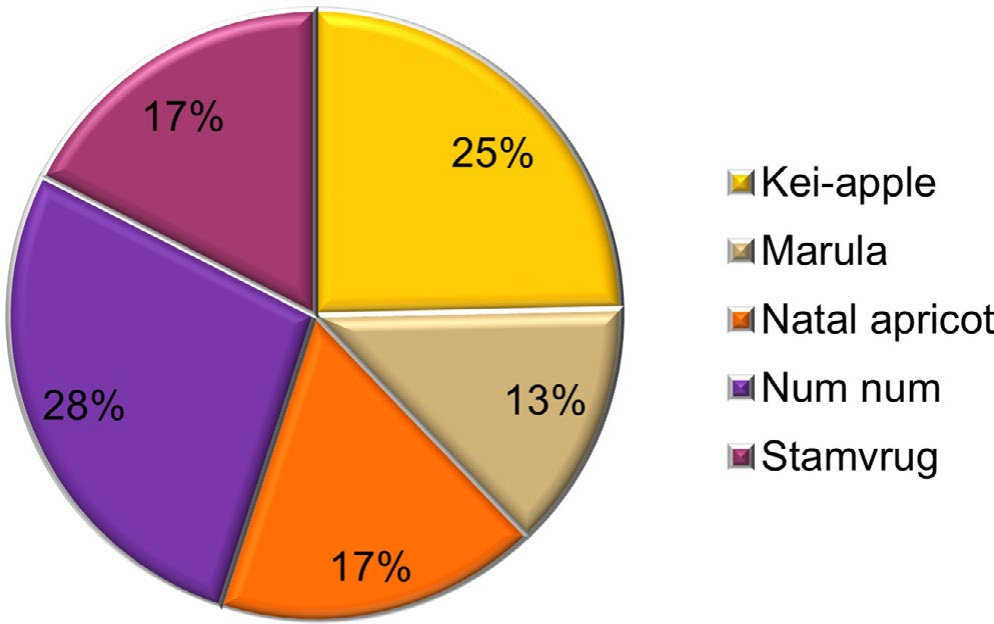
Percentage visual acceptance of indigenous fruit jams as determined by a panel (*n* = 98).

Most consumers preferred the color and fruit image of the num‐num jam (28%), followed by the Kei‐apple jam (25%). The appearance of the marula jam (13%) was the least acceptable (Figure 2). The color and appearance of the num‐num and Kei‐apple jams were the most visually appealing, indicating that consumers may have a stronger preference for purchasing these jams. According to Pandiselvam et al. (2023), food color is the most important aspect in estimating the quality of food and influences the purchasing behavior of consumers.

**FIGURE 3:**
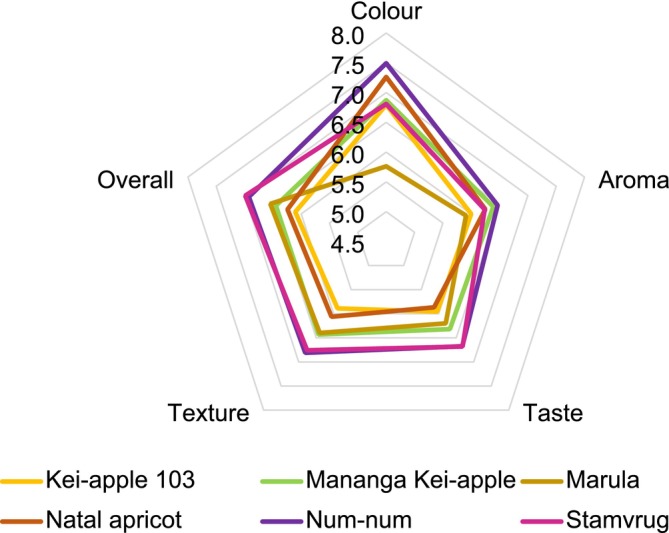
Consumer acceptability for the color, taste, smell, texture, and overall impression of the indigenous fruit jams according to the Hedonic scale.

The preference for the bright‐colored jams of num‐num, Kei‐apple, Natal apricot, and stamvrug among consumers was notably higher compared to marula jam. The marula jam color was significantly less attractive (Table 1), which can be attributed to its L* a* b* values (28.90, 3.10, 14.30) (Table 2). Specifically, the lower a* value indicates a shift from red towards green, resulting in a less appealing hue (Table 2). While there were no significant differences in the aroma, taste, and texture of the jams, the overall impression indicated that the stamvrug and num‐num jams were the favorites (Figure 3; Table 1), with 70% and 63% of the panelists indicating they would buy the product. The intermediate consistency and texture of the jams (not too runny nor too hard) were acceptable. Additionally, the panelists preferred Mananga Kei‐apple jam over the Kei‐apple 103 selection (Table 1). Most of the panelists favored the taste of num‐num and stamvrug jams, which significantly contributed to the overall preference. Results by Udani et al. (2021) also demonstrated that taste was the most important sensory attribute for the overall impression. Aroma plays a vital role in determining the acceptability of a new product. Kei‐apples have a very pleasant and distinct aroma. Mananga Kei‐apple jam was preferred over jam made from the Kei‐apple 103 selection. Augustyn et al. (2018) evaluated 28 Kei‐apple selections and demonstrated that Mananga was distinct from the other selections evaluated by using chemical profiling and genetic analysis. This confirms the importance of various selections of indigenous fruits for size, nutritional value, and processing. Haffner et al. (2002) also emphasized the importance of genetic variation in fruit species.

**TABLE 1:** Color, taste, aroma, texture, and overall impression of the Indigenous fruit jams. Values represent an index according to a 9‐point Hedonic scale.

	Color	Aroma	Taste	Texture	Overall
Kei‐apple 103	6.8^b^	6.0^a^	6.0^a^	5.9^b^	6.1^b^
Mananga Kei‐apple	6.9^b^	6.4^a^	6.3^a^	6.4^ab^	6.4^ab^
Marula	5.8^c^	5.9^a^	6.2^a^	6.4^ab^	6.5^ab^
Natal apricot	7.3^ab^	6.3^a^	5.9^a^	6.1^b^	6.2^b^
Num‐num	7.5^a^	6.5^a^	6.7^a^	6.8^a^	6.9^a^
Stamvrug	6.8^b^	6.2^a^	6.7^a^	6.8^a^	7.0^a^

*Note:* Mean values followed by different superscript letters within each column are significantly different according to Fisher's LSD Test (*p* = 0.05).

**TABLE 2:** L* a* b*, Chroma, and Hue of the indigenous fruit jams stored at 25°C and 35°C for 3 and 6 months.

Storage time (months)/temperature (°C)	Kei‐apple 103	Mananga Kei‐apple	Marula	Natal apricot	Num‐num	Stamvrug
	L*					
Fresh	37.73^a^	35.40^a^	28.90^a^	15.40^a^	9.67^b^	17.43^a^
3/25	34.20^b^	29.20^b^	28.70^b^	7.23^b^	12.83^a^	15.30^c^
3/35	33.20^c^	29.10^b^	27.63^c^	6.43^c^	7.17^d^	14.93^d^
6/25	20.00^d^	18.70^c^	23.90^d^	4.67^d^	3.20^e^	15.77^b^
6/35	18.47^e^	15.10^d^	17.80^e^	4.60^d^	7.73^c^	13.70^e^
	a*					
Fresh	19.20^d^	20.97^c^	3.10^e^	12.50^a^	6.03^b^	13.83^a^
3/25	24.43^a^	22.17^b^	4.10^d^	12.57^a^	4.23^d^	13.80^a^
3/35	22.57^b^	25.30^a^	4.93^c^	8.43^b^	7.67^a^	12.93^b^
6/25	21.77^c^	22.17^b^	7.50^b^	4.43^c^	5.60^c^	11.97^c^
6/35	12.40^e^	13.77^d^	8.70^a^	2.90^d^	5.63^c^	11.20^d^
	***b*** *					
Fresh	60.37^a^	50.53^a^	14.30^b^	5.77^b^	0.37^d^	5.20^e^
3/25	50.23^b^	36.90^c^	13.97^c^	6.17^a^	1.27^a^	7.90^d^
3/35	47.67^c^	45.27^b^	13.33^d^	3.67^c^	1.33^a^	8.53^c^
6/25	30.47^d^	20.50^d^	14.60^a^	2.37^d^	0.50^c^	9.27^b^
6/35	17.53^e^	17.93^e^	13.30^d^	0.50^e^	1.07^b^	10.10^a^
	**Chroma**					
Fresh	63.37^a^	54.70^a^	14.60^c^	13.77^b^	6.03^b^	14.80^d^
3/25	55.83^b^	43.03^c^	14.57^c^	13.97^a^	4.40^e^	15.90^a^
3/35	52.73^c^	51.87^b^	14.23^d^	9.20^c^	7.80^a^	15.50^b^
6/25	37.47^d^	28.57^d^	16.40^a^	5.03^d^	5.60^d^	15.13^c^
6/35	21.47^e^	24.70^e^	15.87^b^	2.90^e^	5.73^c^	15.10^c^
	**Hue**					
Fresh	72.37^a^	67.50^a^	77.83^a^	24.63^c^	3.40^d^	20.60^e^
3/25	64.67^b^	59.00^c^	73.60^b^	26.07^b^	16.40^a^	29.73^d^
3/35	64.03^c^	60.77^b^	69.67^c^	23.53^d^	9.83^b^	33.43^c^
6/25	54.80^d^	56.13^d^	62.63^d^	28.13^a^	5.23^c^	37.70^b^
6/35	54.40^e^	38.97^e^	56.87^e^	10.53^e^	10.50^b^	41.87^a^

*Note:* Mean values followed by different superscript letters within each column are significantly different according to Fisher's LSD Test (*p* = 0.05).

### 3.2 Color

**FIGURE 4:**
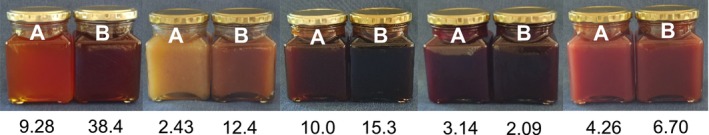
Jam appearance after 6 months of storage at 25°C (A) and 35°C (B). From left to right: Kei‐apple, marula, Natal apricot, num‐num, and stamvrug. Bottom: Delta E color change compared with fresh jams.

Storage temperature was the main factor causing color change in the jams during storage. The difference in color between fresh jams and jams stored at 35°C was the highest in the Kei‐apple jams (Figure 4). The most important color parameters of the indigenous fruit jams during temperature and time storage were L*, b*, and Chroma values. The most significant decrease in lightness (L*) occurred in the Kei‐apple jams stored at 35°C (Table 2). The yellowness of the Kei‐apple jam samples (b*) shifted and declined significantly with time and temperature, with the greatest decline being observed at higher storage temperatures. The hue color changed from initial yellow to reddish tones for the Kei‐apple and marula jams, and from reddish to brown for the stamvrug. The intensity (chroma) of the Kei‐apple and marula jams decreased significantly with storage time and temperature. Statistical analysis showed that the interaction time–temperature factor had a significant effect, with the higher temperature (35°C) having the main influence on the color change (*p* < 0.05).

The color change during storage was most pronounced in both Kei‐apple jams, particularly at higher temperatures. The color of the Kei‐apple jams changed significantly from their fresh state when stored at 35°C. The Delta E values for the Mananga Kei‐apple jam were 25 and 38 after 3 and 6 months of storage, respectively, at this temperature. This color change can be attributed to browning. Good quality jam should retain the color and flavor of the fruit (Rababah et al. 2014). However, storage can influence jam properties (Brandão et al. 2018). The color of food is the first indicator consumers use to determine the perceived quality of food (Pandiselvam et al. 2023; Touati et al. 2014; Udani et al. 2021). Storage conditions of the food are one of the conditions that influence the color (Pandiselvam et al. 2023). Significant color changes (L* a* b* and ΔE) with storage at high temperatures were reported by several authors (Aslanova et al. 2010; Chauhan et al. 2013; Haffner et al. 2002; Touati et al. 2014).

### 3.3 Total Soluble Solids, pH, and Titratable Acids

The pH of the pulp used in this study ranged from 2.6 for Mananga Kei‐apple to 4.0 for marula (Table 3). The pH, titratable acids (TA), total soluble solids (TSS) and TSS/TA ratio of the pulp from the two Kei‐apple selections did not differ significantly. The titratable acidity (TA) of the fruit pulps varied significantly among the different indigenous fruits. The lowest TA was recorded in num‐num pulp (1.34%), while Natal apricot pulp exhibited the highest TA (8.0%). The total soluble solids (TSS) of stamvrug (16.5°Brix) were significantly higher than those of the other fruits, with Kei‐apple selections showing the lowest TSS values. Makumbele et al. (2019) reported similar results on pH (3.10) and TSS (13.51) of num‐num pulp; however, the TA (0.27 g/mL) was significantly lower. The pH value for the marula pulp was similar to the 3.98 obtained by Amarteifio and Mosase (2006). The significantly lower TSS value for Kei‐apple (7.4%) reported by Du Preez et al. (2012) can be attributed to the variation in TA and TSS values for Kei‐apple species.

**TABLE 3:** pH, Titratable acid (TA), Total soluble solids (TSS), TSS/TA ratio, vitamin C (mg/100 g), lycopene total carotene (mg/100 g), Beta‐carotene (mg/100 g), phenolic (mg GAE/g), flavonoid (mg CE/g) and tannin (mg cyanidin chloride/g) of indigenous fruit pulp.

	Kei‐apple 103	Mananga	Marula	Natal apricot	Num‐num	Stamvrug
pH	2.66^c^	2.64^c^	3.96^a^	2.57^c^	3.06^b^	3.16^b^
%TA	5.92^b^	5.69^b^	3.01^c^	8.06^a^	1.34^e^	2.07^d^
TSS	11.87^d^	11.80^d^	13.73^c^	14.33^b^	13.90^c^	16.53^a^
TSS/TA	2.00^d^	2.07^d^	4.73^c^	1.77^d^	10.43^a^	8.07^b^
Vitamin C	0.00^c^	21.08^a^	5.96^b^	4.96^b^	4.93^b^	6.58^b^
Lycopene	6.03^e^	1.87^f^	27.93^a^	22.55^c^	25.39^b^	18.69^d^
Total Carotene	24.23^b^	37.83^a^	21.27^c^	15.06^e^	16.17^d^	9.26^f^
Beta‐carotene	1.26^c^	1.43^bc^	2.42^a^	1.61^b^	0.00^d^	0.00^d^
Phenolic	16.16^b^	12.01^c^	19.25^a^	6.15^d^	11.67^c^	15.83^b^
Flavonoids	6.30^d^	5.20^e^	10.79^a^	4.31^f^	10.26^b^	8.09^c^
Tannins	1.89^d^	1.69^d^	12.74^c^	17.88^b^	22.07^a^	12.46^c^

*Note:* Mean values followed by different superscript letters within each row are significantly different according to Fisher's LSD Test (*p* = 0.05).

The pH values of the indigenous fruit pulp were consistently below 4, and it is, therefore, safe to use for jam‐making as *Clostridium botulinum* is unable to grow at a pH below 4.6 (Odlaug and Pflug 1978). According to Oo and Than (2019), pH is a critical factor in the optimal gelling of jams. No significant differences in pH, TA, TSS, and TSS: TA ratios were observed between the two Kei‐apple selections evaluated. However, previous studies revealed that the TSS: TA ratio of the Mananga fruit was significantly higher than that of the Kei‐apple 103 selection. The TSS values of the Mananga fruit were not exceptionally high, but the TA values were lower in comparison to the other selections, resulting in a high TSS: TA ratio (Augustyn et al. 2018).

The TSS/TA ratios of the “stamvrug” (8.00) and num‐num (10.37) jams were the highest compared to the jams of other fruits. This higher TSS/TA ratio is associated with a more acceptable taste of the jams (Figure 2 and Table 1).

The total soluble solids (TSS) of all the fresh indigenous fruit jams evaluated ranged between 60.1 and 68.6, which falls within the acceptable range for high‐quality jams. TSS levels below 60°Brix may result in a weak gel, while TSS levels above 70°Brix can lead to sugar crystallization, causing an undesirable jam texture (Adeoti et al. 2021).

Storage at 25°C and 35°C for 3 and 6 months did not affect the pH of the jams, except for the “stamvrug” jam (Table 4). The titratable acidity of Mananga Kei‐apple jam decreased by 23.57% over time and with increasing temperature, with the most significant reduction of 21.43% observed after 6 months at 35°C. Conversely, the titratable acidity of the Kei‐apple 103 selection increased under the same conditions. No significant differences were observed for the marula, num‐num, and stamvrug jams.

**TABLE 4:** pH, Titratable acid (TA), Total soluble solids (TSS), and TSS/TA ratio of the indigenous fruit jams stored at 25°C and 35°C for 3 and 6 months.

Storage time (months)/temperature (°C)	Kei‐apple 103	Mananga Kei‐apple	Marula	Natal apricot	Num‐num	Stamvrug
	**pH**					
Fresh	2.83^a^	2.80^a^	3.90^a^	2.93^a^	3.10^a^	3.17^a^
3/25	2.80^a^	2.80^a^	3.93^a^	2.90^a^	3.10^a^	3.20^a^
3/35	2.80^a^	2.80^a^	3.90^a^	2.90^a^	3.07^a^	3.20^a^
6/25	2.60^a^	2.80^a^	3.90^a^	2.90^a^	3.07^a^	3.20^a^
6/35	2.73^a^	2.70^a^	3.90^a^	2.87^a^	3.10^a^	2.93^b^
	**% Titratable acids (TA)**					
Fresh	1.96^b^	2.36^a^	1.54^a^	2.69^ab^	0.98^a^	1.17^a^
3/25	2.12^a^	2.34^a^	2.17^a^	2.60^bc^	1.01^a^	1.18^a^
3/35	1.95^a^	2.26^ab^	1.92^a^	2.74^ab^	0.97^a^	1.21^a^
6/25	2.05^ab^	2.33^a^	2.13^a^	2.93^a^	1.10^a^	1.23^a^
6/35	2.14^a^	2.14^b^	2.21^a^	2.40^c^	1.06^a^	1.21^a^
	**Total soluble solids (TSS)**					
Fresh	68.60^a^	66.10^ab^	60.10^a^	66.20^b^	61.20^c^	60.50^a^
3/25	68.77^a^	66.70^a^	59.00^a^	67.37^ab^	61.37^c^	62.43^a^
3/35	68.43^a^	65.20^cd^	60.83^a^	67.73^ab^	60.53^d^	61.43^a^
6/25	68.97^a^	64.47^d^	60.53^a^	65.93^b^	63.03^a^	63.13^a^
6/35	67.30^b^	65.90^bc^	61.47^a^	69.03^a^	62.13^b^	63.53^a^
	**TSS/TA**					
Fresh	34.95^a^	28.04^b^	38.95^a^	24.64^b^	62.45^a^	51.71^a^
3/25	32.39^b^	28.5^b^	27.15^a^	25.94^b^	60.58^a^	52.91^a^
3/35	35.15^a^	28.89	31.63^a^	24.69^b^	62.21^a^	50.77^a^
6/25	33.64^ab^	28.28^b^	28.38^a^	22.52^c^	57.30^a^	51.33^a^
6/35	31.40^b^	30.08^a^	27.81^a^	28.73^a^	58.78^a^	52.37^a^

*Note:* Mean values followed by different superscript letters within each column are significantly different according to Fisher's LSD Test (*p* = 0.05).

Total soluble solids (TSS) of all the fresh jams were within acceptable values, ranging from 60.5°Brix for stamvrug to 68.6°Brix for Kei‐apple.

Storage at 35°C for 6 months resulted in a significant decrease in TSS in Kei‐apple 103 jams, while TSS remained stable in Mananga Kei‐apple and marula jams over the same period (Table 4). However, an increase in TSS was observed in Natal apricot, num‐num, and stamvrug jams. The TSS/TA ratio of the Kei‐apple 103 selection significantly decreased after 6 months of storage at 35°C compared to the fresh jam, whereas the Mananga selection exhibited a significant increase in the ratio under the same storage conditions (Table 4).

Research by Oo and Than (2019) indicated that the acidity of the mixed fruit jam containing mango and pineapple increased over the first three months and subsequently stabilized. No change in the total soluble solids (TSS) was observed during the six‐month storage period. Singh et al. (2009) reported an increase in acidity after 30 days and a subsequent reduction in acidity between 30 and 60 days of storage of mixed fruit jams. (Mention how this corresponds to your results regarding TSS).

### 3.4 Phytochemical Analysis of the Fruit Pulp

The lycopene, total carotene, flavonoids, phenolics, and tannins differed significantly between all the indigenous fruit pulps (Table 4). Marula contained the highest lycopene (27.93 mg/100 g), followed by num‐num (25.39 mg/100 g) and Natal apricot (22.55 mg/100 g). Mananga Kei‐apple pulp contained significantly more total carotene than the Kei‐apple 103 selection with 37.83 mg/100 g and 24.23 mg/100 g, respectively. The highest flavonoid content (10.79 mg CE/g) was present in the marula pulp, followed by num‐num pulp (10.26 mg CE/g). Marula contained the highest phenolics (19.25 mg GAE/g), Kei‐apple 103 (16.16 mg GAE/g), and Natal apricot contained the least (6.15 mg GAE/g). The tannin content of num‐num was 22.07 mg cyanidin chloride/g, and both Kei‐apple selections contained low tannins of 1.89 mg and 1.69 mg cyanidin chloride/g for the 103 and Mananga selections, respectively.

### 3.5 Phytochemical Composition of the Jam

#### 3.5.1 Vitamin C

High vitamin C levels of 182 mg/100 g and 173 mg/100 g were present in the Kei‐apple 103 and Mananga Kei‐apple jams, respectively. The vitamin C in the Mananga Kei‐apple and marula jams stored at 35°C was significantly reduced and was less affected by storage time. With the Mananga Kei‐apple jam, the vitamin C values declined from 173 mg/100 g to 63.9 mg/100 g and 52.6 mg/100 g after 3‐ and 6‐month storage at 35°C. The vitamin C in the Mananga Kei‐apple and marula jams stored at 35°C was significantly reduced, but was less affected by storage time. Similar findings were reported where a more significant decrease in vitamin C content was observed when apple jams were stored at higher temperatures (Djaoudene et al. 2024).

#### 3.5.2 Lycopene

The highest lycopene content (39.23 g/100 g) was detected in the Natal apricot jams stored at 35°C for three months. The lycopene content of the marula jams decreased from 16.80 mg/100 g for the fresh jams to 9,90 mg/100 g in jams stored at 35°C for 6 months. In both Kei‐apple selections, there was a decrease in lycopene content after 3 months at 25°C. According to Pandiselvam et al. (2023), food undergoes chemical reactions, resulting in the loss of nutrients, polyphenolic compounds, and pigments that affect the color of food. Lycopene is a natural pigment in a non‐provitamin A carotenoid that gives some vegetables and fruits their red to pink colors. It is an antioxidant that protects your body against cell damage. Li et al. (2018) reported a significantly higher decrease in the lycopene content at 37°C than at 25°C. Pathak and Sagar (2023) reported that lycopene is one of the main carotenoids in our diet. Research by Ajmera (2006) concluded that shorter heat treatment can result in better lycopene retention during cooking. However, excessive heat treatment will negatively affect the lycopene content (Jatau et al. 2017).

#### 3.5.3 Carotene

The total carotene content of the Kei‐apple and the Natal apricot jams increased during storage (Table 5). The increase was significantly more for the Kei‐apple 103 and Natal apricot jams stored at 35°C than at 25°C. The highest carotene content was present in the Natal apricot jam (23.37 mg/100 g) stored at 25°C for 3 months. The fresh marula, num‐num, and stamvrug jams contained 7.50 mg/g, 5.17 mg/g, and 4.70 mg/g of carotene, respectively, and the content decreased with storage time and temperature.

**TABLE 5:** Vitamin C, total carotene, Beta‐carotene, phenolic, flavonoid, tannin, and lycopene content of fresh jams or jams stored at 25°C and 35°C for 3 and 6 months.

Storage time (months)/temperature (°C)	Kei‐apple 103	Mananga Kei‐apple	Marula	Natal apricot	Num‐num	Stamvrug
	**Vitamin C (mg/100 g)**					
Fresh	182.10^a^	173.00^a^	116.74^a^	21.00^a^	0.00^a^	0.00^a^
3/25	117.70^c^	159.90^b^	104.00^b^	0.00^c^	0.00^a^	0.00^a^
3/35	116.20^c^	63.90^d^	25.99^e^	0.00^c^	0.00^a^	0.00^a^
6/25	181.30^a^	72.30^c^	93.10^c^	9.53^b^	0.00^a^	0.00^a^
6/35	174.60^b^	52.60^e^	59.70^d^	0.00^c^	0.00^a^	0.00^a^
	**Lycopene (mg/100 g)**					
Fresh	6.40^a^	9.40^c^	16.80^a^	21.80^d^	11.70^b^	11.80^a^
3/25	4.60^a^	8.50^d^	11.70^a^	23.00^c^	11.07^c^	11.40^c^
3/35	4.40^a^	10.70^b^	11.50^a^	39.23^a^	10.40^d^	10.80^d^
6/25	8.70^a^	14.07^a^	11.00^a^	21.70^d^	12.20^a^	11.7
6/35	6.70^a^	7.60^e^	9.90^a^	23.30^b^	11.03^c^	10.43
	**Total carotene (mg/100 g)**					
Fresh	13.33^d^	6.90^e^	7.50^a^	17.40^d^	5.17^a^	4.70^a^
3/25	19.50^b^	8.50^b^	4.83^b^	17.60^c^	4.50^c^	4.40^a^
3/35	19.90^a^	7.90^c^	4.70^c^	23.37^a^	4.10^e^	4.30^a^
6/25	12.10^e^	7.73^d^	4.73^c^	15.10^e^	4.80^b^	4.50^a^
6/35	14.00^c^	14.00^a^	4.00^d^	19.10^b^	4.37^d^	4.10^a^
	**Beta‐carotene (mg/100 g)**					
Fresh	1.20^b^	0.00^a^	0.00^b^	1.30^a^	0.00^b^	0.00^a^
3/25	0.00^c^	0.00^a^	0.00^b^	1.33^a^	0.00^b^	0.00^a^
3/35	1.27^a^	0.00^a^	1.23^a^	1.30^a^	1.23^a^	1.20^a^
6/25	0.00^c^	0.00^a^	0.00^b^	1.33^a^	0.00^b^	0.00^a^
6/35	0.00^c^	0.00^a^	1.20^a^	1.30^a^	0.00^b^	0.00^a^
	**Phenolic (mg GAE/g)**					
Fresh	1.33^b^	2.47^a^	3.03^ab^	1.97^a^	0.97^d^	1.67^c^
3/25	1.57^ab^	1.30^c^	2.20^c^	2.03^a^	1.80^b^	2.53^a^
3/35	1.10^b^	1.83^b^	2.73^b^	1.27^b^	2.07^a^	1.83^b^
6/25	1.20^b^	1.90^b^	1.03^d^	2.07^a^	1.47^c^	1.57^cd^
6/35	2.03^a^	1.33^c^	3.47^a^	1.07^b^	2.03^a^	1.53^d^
	**Flavonoid (mg CE/g)**					
Fresh	0.10^b^	0.37^ab^	1.70^b^	0.70^b^	1.33^b^	0.50^b^
3/25	0.00^c^	0.47^a^	0.07^d^	0.33^d^	0.60^d^	0.83^a^
3/35	0.27^a^	0.27^b^	1.30^c^	0.93^a^	1.67^a^	0.53^b^
6/25	0.10^b^	0.07^c^	0.10^d^	0.13^e^	0.10^e^	0.10^c^
6/35	0.10^b^	0.10^c^	1.90^a^	0.47^c^	1.00^c^	0.60^b^
	**Tannin (mg cyanidin chloride/g)**					
Fresh	1.30^b^	1.47^b^	1.33^d^	5.10^a^	8.03^a^	5.03^c^
3/25	1.67^a^	1.00^cd^	2.30^b^	1.30^d^	4.13^d^	4.10^d^
3/35	0.93^c^	1.83^a^	3.87^a^	4.40^b^	6.63^b^	11.37^a^
6/25	1.60^a^	1.23^bc^	1.80^c^	0.93^d^	6.57^bc^	4.97^c^
6/35	0.93^c^	0.97^d^	3.83^a^	2.93^c^	6.17^c^	6.67

*Note:* Mean values followed by different superscript letters within each column are significantly different according to Fisher's LSD Test (*p* = 0.05).

The carotene content of the marula, num‐num, and “stamvrug” jams decreased with storage time and temperature. The findings of Deng et al. (2022) revealed that higher temperatures and prolonged storage durations resulted in a significant loss of carotenoids, particularly at 35°C. However, the carotene content of the Kei‐apple 103 and Natal apricot jams increased during storage at 35°C.

#### 3.5.4 Phenolics and Flavonoids

The jam processing resulted in a considerable decrease in phenolic and flavonoid content in the jams from all the fruit (Table 5). The phenolic content of marula jam decreased after 3 months of storage and increased after 6 months, with the highest phenolic content of 3.475 mg GAE/g in the jams stored at 35°C. After 6 months of storage, the phenolic content of the num‐num jam increased while the phenolic content of the Natal apricot jams decreased (Table 5). Similar findings were reported for black raspberry jams, with a reduction of 78.9% (Šavikin et al. 2009) and an 89% reduction in Japanese quince fruit preserves (Marat et al. 2025). The processing of Japanese quince also led to a significant reduction in phenolic content within the jams (Marat et al. 2025). During storage time and temperature, the phenolic content of indigenous fruit jams varied considerably. Marula jam exhibited an initial decrease in phenolic content, followed by an increase after 6 months at high temperatures, whereas the phenolic content of Natal apricot jam was negatively impacted under the same conditions. A similar trend was observed in strawberry jams, where an initial reduction in phenolic compounds was followed by an increase until 120 days, where they were similar to the initial phenolic compounds. This might be due to structural changes in phenolics after 30 days (Pineli et al. 2015). Deng et al. (2022) reported that the phenolic content declined with both storage temperature and time. Rababah et al. (2011) found that the total phenolic content significantly decreased in apricot, fig, and orange jams stored at 25°C for 5 months, whereas strawberry and cherry jams retained their phenolic content under similar conditions. Storage time and temperature significantly reduced the phenolic content of apple jam (Djaoudene et al. 2024).

The flavonoid content of the marula decreased after 3 months of storage and increased again after 6 months when stored at 35°C. The highest flavonoid content (1.90 mg CE/g) was observed in the marula jam stored at 35°C for 6 months. In both Natal apricot and num‐num jam, the flavonoids were more affected by temperature than by time. The flavonoid content of the Natal apricot in jams stored at 35°C for 3 months was 0.93 in comparison with 0.47 mg CE/g when stored at 25°C for 3 months. After 3 months of storage, the num‐num jams the flavonoid values of the jams stored at 25°C and 35°C were 1.67 mg CE/g and 1.00 mg CE/g, respectively.

#### 3.5.5 Tannins

Both Kei‐apple selections were low in tannins, ranging from 0.93 to 1.83 mg cyanidin chloride/g per sample across different storage times and temperatures. The tannin content of the marula and stamvrug jam increased after 6 months of storage. The highest tannin content (11.4 mg cyanidin chloride/g) was observed in the stamvrug jam stored at 35°C for 3 months. Natal apricot resulted in a significant decrease when stored at 25°C. The tannin content of the num‐num jams decreased after 3 months of storage at 25°C, followed by an increase after 6 months of storage at 25°C.

#### 3.5.6 Potential of Underutilized Fruits

Underutilized fruits are often recognized as “poor man's crops”, (Kamran et al. 2025). Lesser‐known fruits can be a valuable source of nutrition and the development of healthier products (Adindu‐Linus et al. 2024). Rafique et al. (2023) reported that karonda fruit is an underutilized crop that can be successfully used for flavourful and visually appealing jams containing valuable phytochemical compounds that can be stored without deterioration. Results by Šavikin et al. (2009) and Rababah et al. (2014) also indicated that even though jam‐making reduced phenolics, total anthocyanins, and radical scavenging activity, they were still good sources of antioxidants. According to Omotayo and Aremu (2020), indigenous fruit trees in Africa are valuable sources of food and nutrition security. An increase in the value chain of underutilized fruit trees can contribute to the livelihoods of smallholder farmers, mainly through income generation. Given the limited fruiting season of indigenous fruits, processing plays a crucial role in preserving their availability and maximizing their utilization. According to Nistor et al. (2025), jam processing can change fruits that are not suitable for fresh consumption into functional foods with prolonged storage. There is significant variation in TSS, TA, TSS: TA ratio, and nutritional values between Kei‐apple selections. Kei‐apple selections with a higher TSS: TA ratio can be suitable for fresh consumption, while others are more suitable for processing. Raza et al. (2025) also revealed considerable differences in loquat cultivars and their products, where some cultivars were more suitable for product development and others were suitable for fresh consumption. Additional research on the product development of the more acidic indigenous fruit could explore mixed fruit jam, including commercial fruits. This approach would enhance the taste profile. Ahmad and Zia‐ud‐din Khan (2022) reported that mixed fruit jam was the most acceptable jam when compared to apple jam, guava jam, and citrus marmalade. Proper jam processing techniques help retain essential vitamins and minerals in the fruit and contribute to food security. Fruit products can be enjoyed year‐round, regardless of their natural growing season (Aksay et al. 2018). Value‐adding of these fruits will ensure enhanced nutrition for a longer period (Shinwari and Rao 2018). Processing fruits into jam could generate adequate income for small‐scale processors and contribute to better nutrition and food security (Ndabikunze et al. 2011; Owolade et al. 2016). Amaar et al. (2025) stated that novel product development, including jam production, can create opportunities for local and international markets. Uddin et al. (2024) highlighted that underutilized indigenous fruits have the potential to boost the economy by generating job opportunities within small and medium enterprises. Sosa et al. (2025) emphasized the importance of selection and domestication of wild fruits with desirable sensory and nutritional properties. Currently, indigenous fruits are harvested in the wild. This limits the potential growth of the industry (Omotayo and Aremu 2020). Domestication of indigenous fruit trees will support the commercialisation of products (Kunene et al. 2020; Mapongmetsem et al. 2012; Onomu 2023), it will ensure the conservation of indigenous fruit trees in the wild (Kunene et al. 2020; Omotayo and Aremu 2020), and play a major role in alleviating poverty in rural communities. Increased utilization of these fruits might encourage farmers to cultivate the crops (Ajenifujah‐Solebo and Aina 2011).

## 4 Conclusions

Results from this study indicated that most South African panelists preferred jams with a higher Total soluble solid/Titratable acid ratio, like the num‐num, stamvrug, and marula. Although Kei‐apple fruit is too acidic for fresh consumption, it is suitable for jam‐making. Substantial compositional differences were found among the different fruit species and between different selections. These findings emphasize the need for research on more indigenous fruits. Future product development of indigenous fruit jams could explore mixed fruit variants that combine indigenous and commercial fruits. This approach would enhance both the taste profile and nutritional value of the jams, catering to broader consumer preferences. Processing, storage time, and temperature altered phytochemicals, but phenolics, flavonoids, tannins, lycopene, and tannins were still present in the products that contained these compounds in the fresh state. Jam color stability was greater at 25°C than at 35°C. The study demonstrated that the selected indigenous fruits can be used to make high‐quality indigenous fruit jams and stored at 25°C for up to 6 months with minimal quality degradation. All evaluated indigenous fruit jams exhibit the potential for commercial development and niche market sales. Nevertheless, the technology for producing fruit conserves cannot be universally applied to indigenous fruits, necessitating specific product development research for each fruit. Such development will ensure extended shelf‐life and income generation through value addition.

## Author Contributions

**Karen de Jager:** conceptualization (equal), investigation (lead), visualization (lead), writing – original draft (lead). **Wilma Augustyn:** conceptualization (equal), supervision (equal), writing – review and editing (supporting). **Thierry Regnier:** conceptualization (equal), supervision (equal), writing – review and editing (equal). **Belinda Meiring:** conceptualization (equal), supervision (equal), writing – review and editing (supporting).

## Conflicts of Interest

The authors declare no conflicts of interest.

## Data Availability

The data that support the findings of this study are available on request from the corresponding author.
